# Comparison of One-Year Functional Outcomes and Quality of Life between Posterior Pelvic Ring Fixation and Combined Anterior-Posterior Pelvic Ring Fixation after Lateral Compression (B2 Type) Pelvic Fracture

**DOI:** 10.3390/medicina57030204

**Published:** 2021-02-26

**Authors:** Giedrius Petryla, Valentinas Uvarovas, Rokas Bobina, Jaunius Kurtinaitis, Roma Puronaitė, Giedrius Kvederas, Igoris Šatkauskas

**Affiliations:** 1 Clinic of Rheumatology, Orthopaedics Traumatology and Reconstructive Surgery, Faculty of Medicine, Vilnius University, LT-03101 Vilnius, Lithuania; giedrius.petryla@gmail.com (G.P.); valiusuvarovas@gmail.com (V.U.); jauniusk1@yahoo.com (J.K.); giedrius.kvederas@santa.lt (G.K.); igoris.satkauskas@gmail.com (I.Š.); 2 Centre of Orthopaedics and Traumatology, Republican Vilnius University Hospital, Šiltnamių str. 29, LT-04130 Vilnius, Lithuania; 3 Centre of Informatics and Development, Department of Information Systems, Vilnius University Hospital Santaros Klinikos, LT-08661 Vilnius, Lithuania; romantina@gmail.com; 4 Centre of Orthopaedics and Traumatology, Vilnius University Hospital Santaros Klinikos, LT-08661 Vilnius, Lithuania

**Keywords:** pelvic fracture, posterior pelvic fixation, combined anterior-posterior pelvic fixation, pelvic function, quality of life

## Abstract

*Background and Objectives*: The treatment algorithm of lateral compression B2 type pelvic fractures are still under debate. Some authors advocate conservative treatment, while others recommend surgical approach. The clear indications for isolated anterior or posterior ring fixation or combined anterior-posterior pelvic ring fixation of B2 type fractures remain unclear. The aim of this study was to compare the functional outcomes and quality of life after isolated posterior pelvic ring fixation and combined anterior-posterior pelvic ring fixation for the treatment of B2 pelvic fractures. *Materials and Methods*: Patients aged 18 to 65 years with B2 type pelvic fracture hospitalized in a single trauma centre over a period of 3 years were included in the research. Based on the attending surgeon’s preference, patients were treated with isolated posterior or combined anterior-posterior pelvic fixation. The quality of life and pelvic function were assessed using SF-36 and Majeed questionnaires, respectively. Patients filled in the questionnaires twice: during the first hospitalization (concerning their pre-trauma state—timepoint I) and one-year after the injury (timepoint II). *Results*: A cohort of 32 patients with B2 type pelvic fracture was enrolled in the analysis: 23 (72%) were female and 9 (28%) were male. The mean age was 35.3 ± 11.9 years. In this cohort 13 (41%) patients underwent isolated posterior pelvic ring fixation (group I) and 19 (59%) patients underwent combined anterior-posterior pelvic ring fixation (group II). No statistically significant differences were observed between the groups in both timepoints concerning Majeed, SF-36 PCS and MCS scores. However, in both groups Majeed and SF-36 PCS scores were statistically significantly lower one year after pelvic fracture compared with pre-trauma state, while SF-36 MCS scores did not differ. *Conclusions*: No differences were found in quality of life and functional outcomes between isolated posterior pelvic ring fixation and combined anterior-posterior fixation for the treatment of B2 type pelvic fractures.

## 1. Introduction

According to the AO Foundation/Orthopaedic Trauma Association (AO/OTA) pelvic fracture classification, B2 type pelvic fracture is the most common type of pelvic ring fracture for patients of all ages [1,2]. The data provided by Manson et al. showed that lateral compression (LC) fractures account for 63% of all pelvic fractures [3]. In double-leg stance 60% of pelvic ring stability comes from the posterior structures [4], therefore fixation of the posterior ring should provide sufficient pelvic ring stability required for fracture healing and faster patient mobilization. Nevertheless, B2 fractures are commonly treated with anterior fixation alone [4,5]. Vertical stability of the posterior ring in LC pelvic fractures is maintained by the intact strong posterior ligaments [6,7]. According to the literature, iliosacral screws provide more than 80% of pre-traumatic stability, therefore isolated iliosacral screw fixation for the B2 type pelvic ring fractures is suggested [8,9]. However, Avilucea et al. reported that while LC pelvic fractures after combined posterior and anterior ring fixation healed without displacement, it remained unclear whether additional anterior ring fixation would have prevented subsequent displacement in cases when fractures were fixed only posteriorly [10]. Papakostidis et al. revealed that fixation of all the injured elements of the pelvic ring provided better anatomical results and lower malunion rate, but the clear advantage of functional outcomes was not found [11]. Most authors recommend a non-operative treatment for B2 type pelvic fractures, but studies that compare outcomes are still scarce [12].

Based on the literature, B2 type pelvic fractures with more than 1 cm initial displacement could be fixed posteriorly alone to reduce rotational pelvic fracture instability with lower rates of complications, non-unions [8] and earlier mobilization [13].

Nevertheless, the treatment of displaced lateral compression B2 type pelvic ring injuries is controversial. The clear indications for isolated anterior or posterior ring fixation or combined anterior-posterior pelvic ring fixation of B2 type fractures remain unclear.

The aim of this study was to compare the functional outcomes and quality of life after isolated posterior pelvic ring fixation and combined anterior-posterior pelvic ring fixation for the treatment of B2 type pelvic fractures. Our hypothesis was that acceptable outcomes (Majeed > 70 (good or excellent), SF36 PCS > 45 (same or better than population) would be achieved with posterior pelvic ring fixation alone.

## 2. Materials and Methods

This is a single-centre retrospective cohort study that investigates functional outcomes and quality of life after lateral compression B2 type pelvic fractures for patients treated by isolated posterior ring fixation and combined anterior-posterior pelvic ring fixation.

Patients aged 18 to 65 years with lateral compression B2 type pelvic fracture hospitalized in a single trauma centre over a period of 3 years (January 2016–January 2019) were included in the research. Patients older than 65 years or with pathologic pelvic fractures, pregnant women, patients with mental illness, and with a concomitant acetabular fracture were excluded.

All procedures performed in this study involving human participants were in accordance with the ethical standards of the Vilnius Regional Biomedical Research Ethics Committee (approval No. 158200-16-868-394, 4 November 2016) and with the Helsinki Declaration of 1975, as revised in 2008.

Pelvic computed tomography (CT) and the measurement of fracture dislocation was performed for each patient. Fractures were classified according to AO/OTA pelvic fracture classification using CT scans by two independent senior radiologists. They also performed the measurements of fracture dislocation. Anterior–posterior displacement of the sacrum was measured on the CT axial view by dropping a vertical line from the middle of the S1 body and drawing perpendicular lines through the anterior aspect of each sacroiliac joint at the level of S1. The difference between the lines was recorded as lateral compression displacement.

Personal and clinical data of each patient were collected: gender, age, comorbidities, injury severity score (ISS), type of surgery (isolated posterior or combined anterior-posterior fixation), concomitant injuries and surgeries, length of surgery, surgical complications, length of hospital stay, and mortality.

Complications related to the surgery were considered as follows: wound infections, surgical implant failure, nerve root damage.

Patients were divided into two groups regarding their surgery: group I—isolated posterior pelvic ring fixation and group II—combined anterior-posterior pelvic ring fixation.

Changes in quality of life were assessed using the Physical component summary (PCS) and Mental component summary (MCS) scores of SF-36 [14]. Additionally, functional outcomes were evaluated using Majeed pelvic score. According to Majeed, functional results were graded: ≥85 excellent; 70–84 good; 55–69 fair; <55 poor [15]. Patients completed the questionnaires twice: for the first time during hospitalization (regarding their pre-traumatic condition—timepoint I) and the second time one-year after the injury (regarding their current condition—timepoint II).

No formal surgery protocol for B2 type pelvic fractures was used. Therefore, the surgery technique and the choice of anterior and posterior fixation was based on the attending surgeon’s preference. Full weight-bearing was allowed on the next day after surgical stabilization of the pelvic ring. However, 9 patients were mobilized on the later postoperative days (from 3 to 7 days after surgery) as other fractures or concomitant injuries affected their mobilization. Anticoagulants were administered in both groups for 4 weeks after the injury. On the first day after the surgery, opioid analgesia was administered, and non-steroidal anti-inflammatory drugs were used for further pain management.

The statistical analysis was performed using the IBM SPSS Statistics for Windows, version 23.0 (IBM Corp., Armonk, N.Y., USA). Shapiro-Wilk test was used for data normality analysis. Non-normally distributed data are presented as median (interquartile range—IQR), normally distributed data are presented as mean ± standard deviation (SD). Groups were compared using Chi-square. Independent and related non-parametric samples were compared using Mann-Whitney U and Wilcoxon tests, respectively. Student’s t-test was used as Mann-Whitney U equivalent for parametric data. For correlation analysis, Spearman’s rank correlation coefficient was used. Differences were considered significant at *p* < 0.05.

## 3. Results

A cohort of 32 patients with B2 type pelvic fracture was enrolled in the analysis. There were 27 patients with B2.1, 3 patients with B2.2, and 2 patients with B2.3 pelvic fractures. Out of 32 patients, 23 (72%) were female and 9 (28%) were male. The mean age was 35.3 ± 11.9 years. The median (IQR) of ISS was 17.5 (11.0–18.0). There were 13 (41%) patients in the group I and 19 (59%) patients in the group II. The statistically significant differences between groups were observed concerning surgery time and the duration of hospitalization, that were longer in the group II. No differences were found between groups regarding other characteristics. Collected data are listed in the Table 1.

### 3.1. Quality of Life and Functional Outcomes

**Figure 1:**
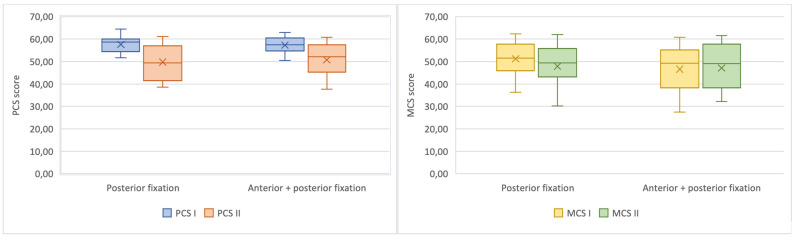
Charts demonstrating the differences of Physical component summary (PCS) (**left**) and Mental component summary (MCS) (**right**) scores of the SF-36 questionnaire between timepoints for patients treated with posterior pelvic ring fixation only and with combined anterior-posterior fixation.

The median (IQR) PCS before the injury was 58.7 (54.2–59.9) points and 49.2 (41.4–56.7) points one-year after the injury. The median (IQR) MCS before the injury was 51.6 (45.1–56.8) points and after one year 49.4 (39.9–55.9) points. There were statistically significant differences between PCS before the injury and one-year after the injury in both groups, however, no differences between the groups were observed. PCS decreased significantly in both groups compared to the state before the injury a year ago (Table 2, Figure 1). No statistically significant differences of MCS were observed neither between timepoints, nor treatment groups (Table 2, Figure 1).

According to the results of Majeed pelvic score questionnaire, pelvic function decreased in both groups one-year after the injury but there were no significant differences between groups. In both groups I and II median (IQR) Majeed pelvic score results changed from 100.0 (100.0–100.0) and 100.0 (100.0–100.0), respectively, before the injury to 88.0 (74.0–95.5) and 87.0 (70.0–96.0), respectively, one-year after pelvic fracture (Table 3). Median Majeed results in both groups before the injury and one-year after pelvic fracture fall into the “excellent” category.

### 3.2. Fracture Dislocation

Pelvic CT scans were used to measure the displacement of the sacral fracture by two radiologists. The median (IQR) of the sacral displacement length in the group I was 7.0 (5.8–9.5) mm compared with 8.5 (6.0–11.0) mm in the group II and no statistically significant difference was observed between the groups (*p* = 0.45). The difference between sacral displacement measurements of two radiologists in the whole cohort was statistically insignificant (*p* = 0.76), however, there was a very strong correlation between the measurements (r = 0.96, *p* < 0.001). More detailed sacral displacement measurements results are presented in the Supplementary Files, Table S1.

### 3.3. Pelvic Surgery Technique

Isolated stabilization of the posterior pelvic ring was achieved using percutaneous iliosacral screw fixation. The C-arm was used for inlet, outlet and lateral views for each patient during the surgery. In the group I fracture stabilization with two iliosacral screws (7.3 mm diameter) were performed for 10 patients (76.9%), while 3 (23.1%) patients underwent fixation with one iliosacral screw (8.0 mm diameter). In the group II for 13 (68.4%) patients percutaneous iliosacral screw fixation was performed in combination with a plate fixation of the anterior pelvic ring. For 3 (15.8%) patients (both patients with B2.3 and one with B2.2 fracture) combined anterior and posterior pelvic ring fixation was achieved with plates. Plate fixation of the posterior pelvic ring was based on the fracture pattern because iliosacral screws would not have provided sufficient stability and was performed through anterior iliac approach. For the other 3 (15.8%) patients anterior and posterior pelvic ring was stabilized using percutaneous screws. There were no cases treated in combination with external fixation. The significant differences between two groups were the surgery time (*p* < 0.001, with statistical power of 99%) and the length of hospital stay (*p* = 0.03, with statistical power of 33%). In the group I the median (IQR) surgery time was 30.0 (25.0–40.0) min, while in the group II the operation lasted 110.0 (65.0–135.0) min. The median (IQR) duration of hospitalization in the group I was 9.0 (6.0–15.0) days in contrast to 15.0 (10.0–22.0) in the group II.

### 3.4. Complications

No surgery-related complications were observed in the group I. However, iatrogenic nerve injury was registered for 3 (15.8%) patients and implant failure was registered for 1 (5.3%) patient in the group II. No wound infections were registered.

## 4. Discussion

We are convinced that good functional outcomes are the most important indicator of the successful treatment of the traumatized patients. The purpose of this study was to evaluate the quality of life and functional outcomes of the patients with lateral compression B2 type pelvic fractures between posterior stabilization alone and a combination of posterior and anterior stabilization. We have found no significant differences in functional outcomes and quality of life between two groups during one-year period. In the group I surgery time and hospital stay were significantly shorter compared with group II. However, while the power analysis revealed that the group sample sizes achieve 99% power to detect the differences between surgery time, there was 33% power only for hospitalization duration.

Based on the literature, posterior sacroiliac arch has the greatest impact on the pelvic ring stability and pelvic pain [1,9,16]. Recently, the minimally invasive percutaneous technique with iliosacral screws has been the most commonly used method for posterior pelvic ring stabilization [17] due to low rate of complications and non-unions [8]. In most of the patients we observed, the posterior pelvic ring was stabilized percutaneously with iliosacral screws (*N* = 13 in the group I; *N* = 16 in the group II) and no surgery-related complications were observed in the group I.

The isolated posterior pelvic ring fixation provides good functional outcomes in lateral compression pelvic fractures [8] same as combined anterior-posterior fixation [18,19]. Posterior pelvic ring stabilization alone may cause less damage, with less blood loss, shorter operative time and lower need for blood transfusions [20]. In addition to these advantages, based on our study, we could speculate that the isolated posterior ring fixation for patients with B2 type fractures may cause shorter hospitalization time. However larger group sample sizes are necessary to prove the difference.

Many studies have examined the quality of life, functional outcomes of pelvic ring injuries, but the problem was the variation in types of fracture, surgical or conservative treatment and because of these reasons we compared our results to clinical series [21].

Quality of life was measured with the SF-36 questionnaire and was compared between two groups before the injury and one-year after the injury. In group I, median (IQR) PCS after one year was 49.3 (41.5–57.0), MCS—49.4 (43.2–55.8). In group II PCS—48.4 (36.1–55.5), MCS—49.1 (39.7–56.3). The quality of life data in our study are similar to the study data of operatively treated patients provided by Höch et al., that compares the quality of life between non-operatively and operatively treated patients with B2.1 type fractures after a mean time of 47 months after trauma [12]. The authors compared the results with German population and showed that for both operatively and non-operatively treated patients PCS scores were lower than the average of German population [12]. Oliver et al. showed much higher SF-36 scores for patients with B types pelvic fractures after 16 months (PCS 68.7 ± 27.6 and MCS 72.2 ± 26.0), nevertheless, the scores were lower than the average of the USA population [22]. Suzuki et al. presented better results of SF-36 (mean PCS was 65.2 (range 22.8–100) points; mean MCS was 67.6 (range, 9.4–100) points) after the mean time of follow-up of 47.2 months, but the patients after pelvic injuries scored lower than the average of Japanese population [23]. It should be mentioned that both of these authors did not distinguish subgroups in their studies, but investigated type B pelvic fractures in general, so it would be inappropriate to compare the data completely. Most of the authors compare quality of life results with the scores of general population. The difference of our study was that we compared PCS and MCS results for the same patient before and after the injury. According to the data of our study, the physical health one-year after the pelvic fracture was significantly lower (with statistical power of 94–100%) in both groups, but no differences were observed concerning the mental health.

Functional outcomes in our study were measured with Majeed pelvic score (group I median (IQR) score 88.0 (74.0–95.5); group II—87.0 (70.0–96.0) after one year). Khaled et al. presented better functional Majeed results of isolated posterior pelvic ring fixation with one and two screws (90.0 ± 11.3 and 89.2 ± 13.6, respectively) for patients with B type pelvic fractures, when the patients had been followed-up for a mean of 37.4 months [21]. According to the research by Van den Bosch et al., the average Majeed score for patients after fixation of both anterior and posterior arches was 78.6 out of 100 [5]. The worse function may have been affected by the use of external fixation for anterior arch. According to the study by Suzuki et al., the average Majeed score was 79.7 (range 30–100) points [23]. The reason for the worse results was the inclusion of patients with C type pelvic fracture. It would be incorrect to compare the results mentioned above due to differences between fracture types, because most of the authors did not classify fractures into subgroups.

Since there are opinions that it is usually sufficient to fix only the anterior pelvic ring after lateral compression pelvic fractures, we reviewed the literature and were able to find one recently published article by Shang et al. that examined the functional outcomes and quality of life of 108 patients using Majeed pelvic score and SF-12 [24]. The follow-up lasted an average of 18.37 (range 7–22) months and the authors reported that the mean SF-12 score was 48.22. The mean Majeed score was 83.47. The data are very similar to the data of our study, so we can assume that the functional outcomes and quality of life do not depend on the type of fixation in the case of B2 type pelvic fracture. Of course, a more accurate assessment requires a prospective randomized study that could confirm or refute this assumption.

There are several limitations to this study. Firstly, this is a single-centre retrospective study. Secondly, no formal surgery protocol was used, and the study was not randomized regarding the surgery method for comparison and evaluation of outcomes after B2 type pelvic fracture. Thirdly, the study populations are relatively small and show some heterogeneity between groups. In addition, only surgery-related complications were registered. The strength of our study is that this is the first study to compare the functional outcomes and quality of life results of isolated posterior pelvic ring fixation with the combined anterior-posterior pelvic ring fixation of B2 type pelvic fracture. Our findings may provide a reference for future randomized controlled trials.

## 5. Conclusions

No differences were found in quality of life and functional outcomes between isolated posterior pelvic ring fixation and combined anterior-posterior fixation for the treatment of B2 type pelvic fractures. Based on the results of this study, treatment of B2 type pelvic fractures with isolated posterior ring fixation is reasonable.

## Figures and Tables

**Table 1:** Comparison of general data between groups. The numbers adjacent to the *p* values in superscript reflect the statistical test used (^1^ Student’s *t*-test, ^2^ Chi-square, ^3^ Mann-Whitney U). The power analysis revealed that the group sample sizes achieve 99% and 33% power to detect the differences of surgery time and hospitalization duration between groups, respectively. *(SD—standard deviation, ISS—injury severity score, IQR—interquartile range).*

	Total	Group I (*N* = 13) Posterior Fixation	Group II (*N* = 19) Posterior-Anterior Fixation	*p* Value
Age (mean ± SD)	35.3 ± 11.9	33.9 ± 11.8	36.3 ± 12.2	0.59 ^1^
Sex (male:female)	9:23	4:9	5:14	0.78 ^2^
Mechanism of accident (*N* (%)):0.75				0.50 ^2^
Motor vehicle accident	15 (46.9%)	6 (46.2%)	9 (47.4%)	0.95 ^2^
Fall from height	12 (37.5%)	6 (46.2%)	6 (31.6%)	0.47 ^2^
Other	5 (15.6%)	1 (7.7%)	4 (21.1%)	0.63 ^2^
Comorbidities (*N* (%))	6 (18.8%)	2 (15.4%)	4 (21.1%)	1.00 ^2^
Surgical complications (*N* (%)):	4 (12.5%)	-	4 (21.1%)	0.13 ^2^
Implant failure	1 (3.1%)	-	1 (5.3%)	1.00 ^2^
Nerve injury	3 (9.4%)	-	3 (15.8%)	0.25 ^2^
Concomitant injuries (*N* (%)):	21 (65.6%)	6 (46.2%)	14 (73.7%)	0.15 ^2^
Cauda equina	1 (3.1%)	-	1 (5.3%)	1.00^2^
Multiple organs injuries (thorax, head, abdomen)	6 (18.8%)	3 (23.1%)	3 (15.8%)	0.67 ^2^
Fracture of one other bone	4 (12.5%)	2 (15.4%)	2 (10.5%)	1.00 ^2^
Fractures of two other bones	6 (18.8%)	1 (7.7%)	5 (26.3%)	0.36 ^2^
Other (wounds, bruises)	3 (9.4%)	-	3 (15.8%)	0.25 ^2^
Other surgery (-ies) (*N* (%))	10 (31.3%)	4 (30.8%)	6 (31.6%)	1.00 ^2^
ISS (median (IQR))	17.5 (11.0–18.0)	18.0 (16.0–18.0)	17.0 (10.0–21.0)	0.54 ^3^
Polytrauma (ISS ≥ 16) (*N* (%))	21 (65.6%)	10 (76.9%)	11 (57.9%)	0.45 ^2^
Surgery time [min] (median (IQR))	62.5 (36.3–120.0)	30.0 (25.0–40.0)	110.0 (65.0–135.0)	<0.001 ^3^
Hospitalization duration [days] (median (IQR))	11.0 (8.3–18.0)	9.0 (6.0–15.0)	15.0 (10.0–22.0)	0.03 ^3^
Sacral fracture dislocation [mm] (median (IQR))	7.5 (6.0–10.4)	7.0 (5.8–9.5)	8.5 (6.0–11.0)	0.45 ^3^

**Table 2:** Differences of Physical component summary (PCS) and Mental component summary (MCS) scores of the SF-36 questionnaire between timepoints and treatment groups. Mann-Whitney U (^1^) and Wilcoxon (^2^) tests were used for comparison of the results. The power analysis revealed that the group sample sizes achieve 100% and 94% power to detect the differences of PCS between timepoints in groups I and II, respectively. *(IQR—interquartile range).*

SF-36	Group I (*N* = 13)	Group II (*N* = 19)	*p* Value
PCS			
before injury (median (IQR)	58.7 (54.4–60.1)	58.6 (54.0–59.9)	0.74 ^1^
one-year after injury (median (IQR)	49.3 (41.5–57.0)	48.4 (36.1–55.5)	0.39 ^1^
*p* value	0.002 ^2^	0.001 ^2^	
MCS			
before injury (median (IQR)	51.5 (45.9–57.9)	52.2 (43.1–56.2)	0.36 ^1^
one-year after injury (median (IQR)	49.4 (43.2–55.8)	49.1 (39.7–56.3)	0.91 ^1^
*p* value	0.21 ^2^	0.64 ^2^	

**Table 3:** Differences of Majeed pelvic score results between timepoints and treatment groups. Mann-Whitney U (^1^) and Wilcoxon (^2^) tests were used for comparison of the results. The power analysis revealed that the group sample sizes achieve 98% and 99% power to detect the differences of Majeed pelvic score results between timepoints in groups I and II, respectively.

Majeed Pelvic Score	Group I (*N* = 13)	Group II (*N* = 19)	*p* Value
Before injury (median (IQR)	100.0 (100.0–100.0)	100.0 (100.0–100.0)	0.36 ^1^
One-year after injury (median (IQR)	88.0 (74.0–95.5)	87.0 (70.0–96.0)	0.86 ^1^
*p* value	0.003 ^2^	0.001 ^2^	

## Data Availability

The data presented in this study are available on request from the corresponding author. The data are not publicly available due to ethical restrictions.

## Supplementary Materials

The following are available online at https://www.mdpi.com/1648-9144/57/3/204/s1, Table S1: title, Detailed data on sacral displacement measurements by two radiologists (A and B) for the whole cohort of 32 patients.
