# Supplementary material for: Comparison of One-Year Functional Outcomes and Quality of Life between Posterior Pelvic Ring Fixation and Combined Anterior-Posterior Pelvic Ring Fixation after Lateral Compression (B2 Type) Pelvic Fracture
Source: Medicina (Kaunas). 2021 Feb 26;57(3):204. doi: 10.3390/medicina57030204 (PMC7996925; doi:10.3390/medicina57030204)
Supplement: Supplementary file 1 [file medicina-57-00204-s001.pdf]

Table S1. Detailed data on sacral displacement measurements by two radiologists (A and B) for the whole cohort of 32 patients

Radiologist	Median	IQR	df
A	8.00	6.00-10.75	31
B	8.00	6.00-10.00	31
Total	7.5	6.0-10.4	63
